# Prevalence Estimation of the *PALB2* Germline Variant in East Asians and Koreans through Population Database Analysis

**DOI:** 10.3390/cancers16193318

**Published:** 2024-09-28

**Authors:** Jong Eun Park, Min-Chae Kang, Taeheon Lee, Eun Hye Cho, Mi-Ae Jang, Dongju Won, Boyoung Park, Chang-Seok Ki, Sun-Young Kong

**Affiliations:** 1 Department of Laboratory Medicine, Hanyang University Guri Hospital, Hanyang University College of Medicine, Guri 11923, Republic of Korea; jongeun820@hanyang.ac.kr; 2 Targeted Therapy Branch, National Cancer Center, Goyang 10498, Republic of Korea; 3 GC Genome, Yongin 16924, Republic of Korea; 4 Department of Laboratory Medicine, Kangbuk Samsung Hospital, Sungkyunkwan University School of Medicine, Seoul 03181, Republic of Korea; 5 Department of Laboratory Medicine and Genetics, Samsung Medical Center, Sungkyunkwan University School of Medicine, Seoul 06351, Republic of Korea; 6 Department of Laboratory Medicine, Yonsei University College of Medicine, Seoul 03722, Republic of Korea; 7 Department of Preventive Medicine, Hanyang University College of Medicine, Seoul 04763, Republic of Korea; 8 Department of Laboratory Medicine, National Cancer Center, Goyang 10498, Republic of Korea; 9 Department of Cancer Biomedical Science, National Cancer Center Graduate School of Cancer Science and Policy, Goyang 10498, Republic of Korea

**Keywords:** *PALB2*, prevalence, East Asian, Korean, gnomAD

## Abstract

**Simple Summary:**

Pathogenic variants in the *PALB2* gene significantly increase the risk of developing breast, ovarian, and pancreatic cancers. However, the prevalence of these mutations in East Asian populations, particularly Koreans, has not been well studied. This research aims to estimate the prevalence of *PALB2* variants in these populations by analyzing large-scale genomic databases. Understanding the frequency of *PALB2* variants can help in identifying individuals at higher risk for these cancers and guide the development of targeted screening and prevention strategies. The findings from this study provide valuable reference data that can enhance genetic counseling and improve cancer risk management in East Asian populations, ultimately contributing to better health outcomes in these communities.

**Abstract:**

*PALB2* is a tumor suppressor gene. Heterozygous germline pathogenic variants of *PALB2* significantly increase the lifetime risk of breast cancer and moderately increase the risk of ovarian and pancreatic cancers. This study analyzed the estimated prevalence of *PALB2* variants globally, focusing on East Asian and Korean populations, where limited data were previously available. We examined 125,748 exomes from the Genome Aggregation Database (gnomAD), including 9197 East Asians, and additional data from 5305 individuals in the Korean Variant Archive and 1722 in the Korean Reference Genome Database. All *PALB2* variants were interpreted according to guidelines from the American College of Medical Genetics and Genomics and the Clinical Genome Resource. The global prevalence of *PALB2* variants was 0.18%, with the highest prevalence in Finnish populations (0.41%) and the lowest in Ashkenazi Jewish populations (0.04%). East Asians had a prevalence of 0.09%. By combining data from Korean genome databases and gnomAD totaling 8936 individuals, the overall prevalence of *PALB2* variants in the Korean population was determined to be 0.13%. This study is the first comprehensive investigation of *PALB2* variant prevalence in East Asians and Koreans using gnomAD and Korean genome databases. These findings provide essential reference data for future research and highlight the importance of region-specific genetic studies that will inform genetic counseling and hereditary cancer risk management.

## 1. Introduction

*PALB2* (partner and localizer of BRCA2) is a tumor suppressor gene crucial for repairing DNA double-strand breaks via the homologous recombination pathway [1]. The PALB2 protein interacts with both BRCA1 and BRCA2, forming an essential part of the BRCA complex (BRCA1-PALB2-BRCA2-RAD51), which maintains genomic stability. Defects in this pathway are linked to cancer development [2]. Biallelic germline loss-of-function mutations in *PALB2* cause Fanconi anemia [3]. In contrast, heterozygous germline pathogenic variants in *PALB2* significantly increase the lifetime risk of breast cancer and moderately increase the risks of ovarian and pancreatic cancers [4,5,6].

The prevalence of germline pathogenic variants in *PALB2* among breast cancer patients has been reported to range from approximately 0.23–2.65% [7,8]. The estimated lifetime risk of breast cancer for female carriers of *PALB2* variants is 53% by age 80 (95% confidence interval, 44–63%) [5]. In control populations, the prevalence of *PALB2* variants is reported to be between 0.0% and 0.75% [4,9]. Most studies have been conducted in Western populations, with limited data on East Asians, highlighting the need for further region-specific research.

Although the spectrum of pathogenic *PALB2* variants is diverse, studies on patients with breast, ovarian, and pancreatic cancers have identified the top five most frequently reported pathogenic variants as c.509_510del;p.(Arg170IlefsTer14), c.3113G>A;p.(Trp1038Ter), c.1592del;p.(Leu531CysfsTer30), c.172_175del;p.(Gln60ArgfsTer7), and c.1240C>T;p.(Arg414Ter), which account for 57.3% of all cases [10]. Notably, the c.1592del;p.(Leu531CysfsTer30) variant is recognized as a founder mutation in approximately 1% of Finnish breast cancer patients [8].

According to the latest National Comprehensive Cancer Network Clinical Practice Guidelines in Oncology for Genetic/Familial High-Risk Assessment: Breast, Ovarian, and Pancreatic (Version 3.2024—February 12, 2024) [11], genetic testing for *PALB2* variants, along with *BRCA1/2*, is recommended for patients with suspected hereditary breast, ovarian, or pancreatic cancer. Individuals with pathogenic *PALB2* variants are advised to follow breast cancer risk management protocols similar to those for individuals with *BRCA1/2* variants. Additionally, carriers of pathogenic *PALB2* variants may consider risk-reducing salpingo-oophorectomy between the ages of 45 and 50 to lower the risk of ovarian cancer.

The gnomAD v2.1.1 database, containing 125,748 exomes with 9197 from East Asian, is a comprehensive global genomic database [12]. Additionally, the Korean Variant Archive (KOVA) provides a reference for genetic variation specific to the Korean population, containing data from 1.896 whole-genome sequencing and 3409 whole exome sequencing [13]. Another major resource, the Korean Reference Genome Database (KRGDB), consists of whole-genome sequencing data from 1722 Koreans [14].

These genomic databases, which encompass diverse ethnic groups, are crucial for estimating the prevalence of *PALB2* variants. In our study, we used the 2015 guidelines from the American College of Medical Genetics and Genomics and the Association for Molecular Pathology guidelines (ACMG/AMP guidelines) to analyze *PALB2* variants within these databases [15]. Our main objective was to assess the global prevalence of *PALB2* variants, with a special focus on East Asian populations that have been underrepresented in previous research.

## 2. Materials and Methods

### 2.1. Population Database

We obtained *PALB2* gene data from gnomAD (v2.1.1), accessible at https://gnomad.broadinstitute.org/ (access on 10 October 2021). Our analysis covered 125,748 exomes from diverse populations, including 9197 East Asians, 8.128 African/African-Americans, 17,296 Latino/Admixed Americans, 5.040 Ashkenazi Jewish, 10,824 Finnish, 56,885 non-Finnish Europeans, 15,308 South Asians, and 3070 from other (population not assigned) populations. Within the East Asian cohort, there were 1909 Koreans, 76 Japanese, and 7212 individuals of other East Asian ancestries. We excluded variants marked with “InbreedingCoeff”, “AC0”, or “RF” quality control filters in gnomAD.

For Korean-specific data, we used the KOVA database, which includes information from 5305 Koreans (https://www.kobic.re.kr/kova/, accessed on 6 November 2023), and the KRGDB, which contains whole-genome sequencing data from 1722 Koreans (http://coda.nih.go.kr/coda/KRGDB/index.jsp, accessed on 25 September 2021).

### 2.2. Classification and Statistical Analysis of PALB2 Variants

All *PALB2* variants were interpreted according to the ACMG/AMP guidelines and the Sequence Variant Interpretation guidelines from ClinGen (https://clinicalgenome.org/working-groups/sequence-variant-interpretation/, accessed on 20 March 2024). These guidelines categorize variants into five groups: pathogenic variant (PV), likely pathogenic variant (LPV), variants of uncertain significance, likely benign variant, and benign variant. In silico prediction of variant pathogenicity was performed using REVEL [16] and SpliceAI [17]. The threshold range for REVEL was determined based on the criteria suggested by Pejaver et al. [18], and a SpliceAI Δ score ≥ 0.2 was applied as the threshold for spliceogenicity [19].

We compared all identified *PALB2* variants in population databases with previously characterized disease-causing variants from ClinVar and the Human Gene Mutation Database (HGMD). ClinVar (https://www.ncbi.nlm.nih.gov/clinvar/, accessed on 21 March 2024) is an open-access repository of variant classifications provided by a variety of sources, including clinical laboratories, research institutions, and expert panels. The HGMD professional database (http://www.hgmd.org/, release 2023.04) is a comprehensive collection of germline variants, categorized into six groups. Our primary focus was on disease-causing mutations (DM).

### 2.3. Prevalence Estimation of PALB2 Variant

The prevalence of *PALB2* variant was determined using population databases. For the prevalence analysis, we included variants classified as PV and LPV according to the ACMG/AMP guidelines, PV and LPV interpretations from ClinVar, as well as DM entries from HGMD. To estimate the prevalence of *PALB2* variants, we considered the autosomal dominant inheritance pattern. The prevalence was estimated using the Hardy-Weinberg equilibrium principle (1 = p² + 2pq + q²), where p represents the major allele (non-disease) and q represents the minor allele (disease). In the case of autosomal dominant inheritance, carriers are represented by 2pq. We predicted the estimated disease prevalence using 2pq. Statistical analyses were conducted using R version 4.3.2, and 95% confidence intervals were computed for each value. This study was approved by the Institutional Review Board of Hanyang University Guri Hospital (approval code is IRB No. HYUH 2021-06-29; granted on 15 July 2021) and was conducted in accordance with the Declaration of Helsinki.

## 3. Results

We conducted an analysis of 125,748 exomes from the gnomAD database, focusing specifically on 9197 exomes from East Asian populations, with an emphasis on variants within the *PALB2* gene. The variants were classified according to ACMG/AMP guidelines and cross-referenced with ClinVar and HGMD, two widely used disease classification databases (Table 1).

According to the ACMG/AMP guidelines, the global prevalence of *PALB2* variants was estimated to be 0.18%. The estimated prevalence exhibited significant variation among different populations. Finnish populations had the highest prevalence at 0.41%, while Ashkenazi Jewish populations had the lowest at 0.04%. East Asians showed the second lowest prevalence among the studied groups, at 0.09%. ClinVar data indicated a global prevalence for *PALB2* variants of 0.17%. According to HGMD data, the overall prevalence for *PALB2* variants was higher, at 0.22%.

In the gnomAD database, the prevalence of *PALB2* variants among Koreans was reported to be 0.10% (Table 2). Additionally, analysis based on the ACMG/AMP guidelines showed a prevalence of *PALB2* variants of 0.19% in the KOVA database, which includes data from 5305 individuals. No *PALB2* variant was observed in the KRGDB database, which includes 1722 individuals. By combining data from gnomAD, KOVA, and KRGDB, totaling 8936 individuals, the overall prevalence of *PALB2* variants in the Korean population was determined to be 0.13%.

**Figure 1:**
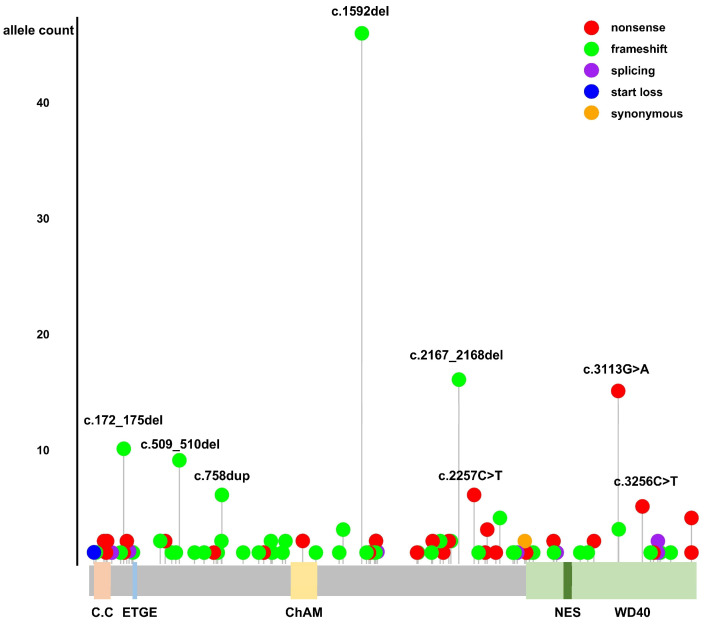
Schematic representation of pathogenic and likely pathogenic variants in the *PALB2* gene from gnomAD, and structural motifs and functional domains of PALB2. C.C.: coiled-coil motif; ETGE motif; ChAM: chromatin association motif; WD40: WD40 repeat; NES: nuclear export sequence.

The summary in Supplementary Table S1 details the PV and LPV identified in the *PALB2* gene, classified according to ACMG/AMP guidelines, within the gnomAD database. Figure 1 provides a schematic representation of these pathogenic and likely pathogenic variants, along with the structural motifs and functional domains of the *PALB2* gene. The c.1592del;p.(Leu531CysfsTer30) variant was the most prevalent globally, identified in 46 alleles. This variant was observed across various ethnic groups, with a notable presence in the European (Finnish) population, but was absent in East Asians. The second-most-frequently observed variant, c.2167_2168del;p.(Met723ValfsTer21), was found in 16 alleles, predominantly in individuals of Latino ancestry, but was not observed in East Asians. Further analysis comparing PV/LPV variants in East Asians with those from other ethnic groups showed notable differences. Except for the c.1050_1053del;p.(Thr351ArgfsTer4) variant, the other variants were exclusively found in East Asian populations. Variants classified as PV/LPV in ClinVar and DM variants listed in HGMD are detailed in Table S2 and Table S3, respectively.

The summary of PV/LPVs found in the Korean database is presented in Table S4. In gnomAD, the variant c.2968G>T;p.(Glu990Ter), which is the most common in East Asians, was not identified in Koreans. Conversely, the c.1048C>T;p.(Gln350Ter) variant was most frequently observed in Koreans. Compared to the total gnomAD population, apart from the c.2167_2168del;p.(Met723ValfsTer21) variant, all variants identified in Koreans were not detected in other ethnic groups

## 4. Discussion

The findings of this study significantly enhanced our understanding of the prevalence and spectrum of *PALB2* pathogenic variants across different populations, with a special focus on East Asians and Koreans. The use of extensive genomic databases, including gnomAD, KOVA, and KRGDB, allowed for a comprehensive analysis, revealing notable differences in variant prevalence among ethnic groups.

Our study determined that the global prevalence of *PALB2* pathogenic variants was approximately 0.18%, with significant variation across populations. Finnish populations showed the highest prevalence at 0.41%, while Ashkenazi Jewish populations had the lowest at 0.04%. East Asians exhibited a relatively low prevalence at 0.09%, with Koreans showing a prevalence of 0.13% when data from gnomAD, KOVA, and KRGDB were combined.

Comparing our results with the case-control study reported by Girard et al., which indicated a control *PALB2* variant prevalence of 0.75% [9], there is a noticeable difference. However, reassessment of the variants identified in that study using the ACMG/AMP guidelines resulted in a control *PALB2* prevalence of 0.25%, which was similar to or slightly higher than the prevalence reported in our study.

In this study, the variants among the PV/LPVs of the *PALB2* gene in the gnomAD database were found in the following order: c.1592del;p.(Leu531CysfsTer30), c.2167_2168del;p.(Met723ValfsTer21), c.3113G>A;p.(Trp1038Ter), c.172_175del;p.(Gln60ArgfsTer7), and c.509_510del;p.(Arg170IlefsTer14). These results are consistent with a previous large-scale systematic review of breast, ovarian, and pancreatic cancers [10], which reported the same top-five *PALB2* variants, with the exception of c.2167_2168del;p.(Met723ValfsTer21). This suggests that genomic database studies can reliably predict prevalent variants in real patients, providing valuable insights for genetic counseling and risk assessment. However, among the 10 *PALB2* variants found in the Korean genomic database, c.1048C>T;p.(Gln350Ter) and c.3267_3268del;p.(Phe1090SerfsTer6) were consistent with two Korean breast cancer patients among Korean breast, ovarian, and pancreatic ductal adenocarcinoma patients [20,21,22,23,24]. This suggests some uncertainty due to the small number of patients in certain ethnic groups and highlights the need for further studies.

To further investigate the variant spectrum, we compared the 10 variants identified in the Korean database with large-scale databases from China (15,068 genomes) and Japan (59,940 genomes) [25,26,27]. Of the 10 variants, only 2 (c.2167_2168del and c.2834+2T>C) were found in the Japanese population, and none were identified in the Chinese population. The variant spectrum found in the Korean population is distinct, not only when compared to other ethnic groups but also within East Asia itself.

The ACMG has updated its guidelines to include *PALB2*, along with *BRCA1/2*, in the ACMG SF v3.2 list for reporting secondary findings from clinical exome and genome sequencing [28]. Additionally, population-based *BRCA1/BRCA2/RAD51C/RAD51D/BRIP1/PALB2* testing has been found to be more cost-effective than strategies based on clinical criteria or family history based *BRCA1/BRCA2/RAD51C/RAD51D/BRIP1/PALB2* testing [29]. It is crucial to evaluate *PALB2* variants during genetic testing, particularly for individuals of European (Finnish) descent, who had the highest carrier frequency in this study. Research indicates that individuals with pathogenic *PALB2* variants have a lifetime breast cancer risk of up to 53% by age 80 [5]. Therefore, implementing a comprehensive surveillance plan, including regular breast cancer screening and considering risk reduction strategies such as prophylactic surgery, is essential [11].

According to data retrieved from the Korea Statistical Information Service (http://kosis.kr/, accessed 3 June 2024), the total population of South Korea as of 2024 is 51.8 million. In this study, among 8936 individuals from the genomic database, the prevalence of *PALB2* PV/LPV was 0.13%. Given this prevalence, it is estimated that approximately 67,000 individuals in Korea carry *PALB2* PV/LPV variants. According to a Korean BRCA1/2 negative breast cancer cohort study, it was confirmed that 2.43% (17/700) of BRCA1/2 negative breast cancers were caused by *PALB2* variants [23]. In addition, *PALB2* variants were identified in one case each in Korean ovarian cancer and pancreatic cancer cohorts [22,24]. However, genetic counseling for patients with *PALB2* variants and their families remains insufficient in Korea. Since *PALB2* variants are inherited in an autosomal dominant, there is a 50% chance that other family members may carry the same variant. Therefore, appropriate prevention and treatment strategies for *PALB2* related cancer will also be needed in Koreans.

This study has several limitations. First, large deletions or insertions (defined as those over 50 base pairs and often spanning multiple exons) within the *PALB2* gene were not included in the analysis, which could result in an underestimation of the actual prevalence of *PALB2* variants. Previous studies have reported the presence of *PALB2* deletions or insertions including Koreans [6,23,30], but such variants were not detectable in our current dataset. Second, the prevalence estimates were derived solely from genes registered in genomic databases, which introduces potential inaccuracies. This approach does not incorporate detailed clinical information from patients, and as additional evidence becomes available, some variants currently classified as variants of uncertain significance (VUS) may be reclassified as PV/LPV, while conversely, PV/LPV may be reclassified as VUS. Moreover, since the penetrance of *PALB2* is not 100%, the actual number of patients expressing the phenotype may be lower than the predicted prevalence. This discrepancy underscores the importance of integrating comprehensive clinical data with genomic information to improve the accuracy of prevalence estimates and variant interpretation.

Despite these limitations, this study effectively estimated the prevalence of *PALB2* variants across various ethnic groups. Notably, this is the most extensive study conducted on East Asians, particularly Koreans, to analyze the *PALB2* gene. Consequently, this research provides a more accurate prediction of carrier frequency and incidence among both East Asians and Koreans. Given the recent advancements in surveillance and treatment options for *PALB2*-associated cancers, identifying carriers of *PALB2* variants is essential for effective management and intervention.

## 5. Conclusions

This study is the first comprehensive investigation of *PALB2* variant prevalence in East Asians and Koreans using gnomAD and the Korean genome database. We found that East Asians have the second-lowest prevalence of *PALB2* variants among various ethnic groups, with significant differences in variant distributions compared to other populations. These findings provide essential reference data for future research and highlight the importance of region-specific genetic studies in enhancing genetic counseling and managing hereditary cancer risks.

## Figures and Tables

**Table 1:** Estimated prevalence of *PALB2* variants in population database.

	Total Alleles (*n*)	Estimated Prevalence (%)	One Carrier in Every (*n*)
Total (*n* = 125,748)			
ACMG/AMP (PV/LPV)	227	0.18 (0.16–0.21)	554 (485–632)
ClinVar (PV/LPV)	214	0.17 (0.15–0.19)	588 (513–673)
HGMD (DM)	277	0.22 (0.20–0.25)	454 (403–512)
East Asian (*n* = 9.197)			
ACMG/AMP (PV/LPV)	8	0.09 (0.04–0.18)	1150 (559–2472)
ClinVar (PV/LPV)	7	0.08 (0.03–0.16)	1314 (609–2998)
HGMD (DM)	14	0.15 (0.09–0.26)	657 (381–1154)
African (*n* = 8128)			
ACMG/AMP (PV/LPV)	16	0.20 (0.12–0.33)	508 (306–858)
ClinVar (PV/LPV)	16	0.20 (0.12–0.33)	508 (306–858)
HGMD (DM)	17	0.21 (0.13–0.34)	478 (292–794)
Latino (*n* = 17,296)			
ACMG/AMP (PV/LPV)	29	0.17 (0.11–0.24)	596 (410–874)
ClinVar (PV/LPV)	28	0.16 (0.11–0.24)	618 (421–912)
HGMD (DM)	39	0.23 (0.16–0.31)	443 (321–615)
Ashkenazi Jewish (*n* = 5040)			
ACMG/AMP (PV/LPV)	2	0.04 (0.01–0.16)	2520 (625–14,548)
ClinVar (PV/LPV)	1	0.02 (0.00–0.13)	5040 (777–96,551)
HGMD (DM)	2	0.04 (0.01–0.16)	2520 (625–14,548)
European (Finnish) (*n* = 10,824)			
ACMG/AMP (PV/LPV)	44	0.41 (0.30–0.55)	246 (182–334)
ClinVar (PV/LPV)	44	0.41 (0.30–0.55)	246 (182–334)
HGMD (DM)	44	0.41 (0.30–0.55)	246 (182–334)
European (non-Finnish) (*n* = 56,885)			
ACMG/AMP (PV/LPV)	105	0.18 (0.15–0.22)	542 (446–659)
ClinVar (PV/LPV)	98	0.17 (0.14–0.21)	580 (474–711)
HGMD (DM)	137	0.24 (0.20–0.29)	415 (350–493)
South Asian (*n* = 15,308)			
ACMG/AMP (PV/LPV)	15	0.10 (0.06–0.17)	1021 (604–1756)
ClinVar (PV/LPV)	12	0.08 (0.04–0.14)	1276 (709–2354)
HGMD (DM)	14	0.09 (0.05–0.16)	1093 (635–1921)
Other (*n* = 3070)			
ACMG/AMP (PV/LPV)	8	0.26 (0.12–0.53)	384 (187–825)
ClinVar (PV/LPV)	8	0.26 (0.12–0.53)	384 (187–825)
HGMD (DM)	10	0.33 (0.17–0.62)	307 (161–604)

95% CI, 95% confidence interval; ACMG/AMP, 2015 American College of Medical Genetics and Genomics and the Association for Molecular Pathology guideline; DM, disease-causing variant; gnomAD, Genome Aggregation Database; HGMD, Human Gene Mutation Database; LPV, likely pathogenic variant; NA, not applicable; PV, pathogenic variant.

**Table 2:** Estimated prevalence of *PALB2* variants in East Asian and Korean population databases.

	Total Alleles (*n*)	Estimated Prevalence (%)	One Carrier in Every (*n*)
gnomAD East Asian exomes (*n* = 9197)			
ACMG/AMP (PV/LPV)	8	0.09 (0.04–0.18)	1150 (559–2472)
ClinVar (PV/LPV)	7	0.08 (0.03–0.16)	1314 (609–2998)
HGMD (DM)	14	0.15 (0.09–0.26)	657 (381–1154)
gnomAD Korean exomes (*n* = 1909)			
ACMG/AMP (PV/LPV)	2	0.10 (0.02–0.42)	954 (237–5510)
ClinVar (PV/LPV)	1	0.05 (0.00–0.34)	1909 (295–36,570)
HGMD (DM)	0	0.00 (0.00–0.25)	NA (399–NA)
gnomAD Japanese exomes (*n* = 76)			
ACMG/AMP (PV/LPV)	0	0.00 (0.00–6.00)	NA (17–NA)
ClinVar (PV/LPV)	0	0.00 (0.00–6.00)	NA (17–NA)
HGMD (DM)	0	0.00 (0.00–6.00)	NA (17–NA)
gnomAD Other East Asian exomes (*n* = 7212)			
ACMG/AMP (PV/LPV)	6	0.08 (0.03–0.19)	1202 (524–2957)
ClinVar (PV/LPV)	6	0.08 (0.03–0.19)	1202 (524–2957)
HGMD (DM)	14	0.19 (0.11–0.33)	515 (299–905)
All Korean (*n* = 8936)			
ACMG/AMP (PV/LPV)	12	0.13 (0.07–0.24)	745 (414–1374)
ClinVar (PV/LPV)	11	0.12 (0.06–0.23)	812 (440–1544)
HGMD (DM)	6	0.07 (0.03–0.15)	1489 (649–3663)
gnomAD Korean exomes (*n* = 1909)			
ACMG/AMP (PV/LPV)	2	0.10 (0.02–0.42)	954 (237–5510)
ClinVar (PV/LPV)	1	0.05 (0.00–0.34)	1909 (295–36,570)
HGMD (DM)	0	0.00 (0.00–0.25)	NA (399–NA)
KOVA (*n* = 5305)			
ACMG/AMP (PV/LPV)	10	0.19 (0.10–0.36)	531 (279–1044)
ClinVar (PV/LPV)	10	0.19 (0.10–0.36)	531 (279–1044)
HGMD (DM)	6	0.11 (0.05–0.26)	884 (386–2175)
KRGDB (*n* = 1722)			
ACMG/AMP (PV/LPV)	0	0.00 (0.00–0.28)	NA (360–NA)
ClinVar (PV/LPV)	0	0.00 (0.00–0.28)	NA (360–NA)
HGMD (DM)	0	0.00 (0.00–0.28)	NA (360 –NA)

95% CI, 95% confidence interval; ACMG/AMP, 2015 American College of Medical Genetics and Genomics and the Association for Molecular Pathology guideline; DM, disease-causing variant; gnomAD, Genome Aggregation Database; HGMD, Human Gene Mutation Database; KOVA, Korean Variant Archive; KRGDB, Korean Reference Genome Database; LPV, likely pathogenic variant; NA, not applicable; PV, pathogenic variant.

## Data Availability

The data presented in this study are available in this article.

## Supplementary Materials

The following supporting information can be downloaded at: https://www.mdpi.com/article/10.3390/cancers16193318/s1, Table S1: Pathogenic and likely pathogenic variants classified according to the 2015 American College of Medical Genetics and Genomics and Association for Molecular Pathology guidelines in gnomAD; Table S2: Pathogenic and likely pathogenic variants in ClinVar from gnomAD; Table S3: Disease-causing mutations in the Human Gene Mutation Database (HGMD) from gnomAD; Table S4: Pathogenic and likely pathogenic variants in Korean databases classified according to the ACMG/AMP guidelines.
