# Supplementary material for: Prevalence Estimation of the PALB2 Germline Variant in East Asians and Koreans through Population Database Analysis
Source: Cancers (Basel). 2024 Sep 28;16(19):3318. doi: 10.3390/cancers16193318 (PMC11476084; doi:10.3390/cancers16193318)
Supplement: Supplementary file 1 [file cancers-16-03318-s001.zip › cancers-3195289-supplementary.pdf]

Table S1. Pathogenic and likely pathogenic variants classified according to the 2015 American College of Medical Genetics and Genomics and Association for Molecular Pathology guidelines in gnomAD.

Nucleotide Change	Amino Acid Change	gnomAD allele frequency									ACMG/AMP evidences
		Total (*n* = 125,748)	East Asian (*n* = 9197)	African (*n* = 8128)	Latino (*n* = 17,296)	Ashkenazi Jewish (*n* = 5040)	European (Finnish) (*n* = 10,824)	European (non-Finnish) (*n* = 56,885)	South Asian (*n* = 15,308)	Other (*n* = 3070)	
c.1A>G	p.(Met1?)	4.06.E-06	0	0	0	0	0	0	0	1.66.E-04	PVS1_Moderate, PS1, PM2_Supporting, PP1
c.2T>C	p.(Met1?)	4.06.E-06	0	0	0	0	0	9.04.E-06	0	0	PVS1_Moderate, PS1, PM2_Supporting
c.12dup	p.(Pro5SerfsTer8)	4.04.E-06	0	0	0	0	0	0	3.28.E-05	0	PVS1, PM2_Supporting
c.62T>G	p.(Leu21Ter)	7.95.E-06	0	0	5.78.E-05	0	0	0	0	0	PVS1, PM2_Supporting
c.73A>T	p.(Lys25Ter)	3.98.E-06	0	0	0	0	0	0	3.27.E-05	0	PVS1, PM2_Supporting
c.79G>T	p.(Glu27Ter)	7.95.E-06	0	1.23.E-04	0	0	0	0	0	0	PVS1, PM2_Supporting
c.109-12T>A	p.(?)	3.98.E-06	0	0	0	0	0	8.79.E-06	0	0	PS1_Supporting, PS3_Moderate, PM2_Supporting, PP1, PP3
c.162del	p.(Glu54AspfsTer14)	3.98.E-06	0	0	0	0	0	8.79.E-06	0	0	PVS1, PM2_Supporting
c.172_175del	p.(Gln60ArgfsTer7)	3.98.E-05	0	0	0	9.92.E-05	0	7.91.E-05	0	0	PVS1, PP1
c.178C>T	p.(Gln60Ter)	3.98.E-06	0	0	0	0	0	8.79.E-06	0	0	PVS1, PM2_Supporting

c.196C>T	p.(Gln66Ter)	7.95.E-06	0	0	0	0	0	1.76.E-05	0	0	PVS1, PM2_Supporting
c.212-2A>G	p.(?)	4.52.E-06	0	0	0	0	0	1.02.E-05	0	0	PVS1_Strong, PM2_Supporting, PP1
c.226del	p.(Ile76TyrfsTer101)	4.09.E-06	0	6.31.E-05	0	0	0	0	0	0	PVS1, PM2_Supporting
c.232_233del	p.(Val78LeufsTer2)	4.04.E-06	0	6.23.E-05	0	0	0	0	0	0	PVS1, PM2_Supporting
c.395del	p.(Val132AlafsTer45)	7.98.E-06	0	0	0	0	0	1.76.E-05	0	0	PVS1, PM2_Supporting
c.424A>T	p.(Lys142Ter)	7.96.E-06	0	0	0	0	0	1.76.E-05	0	0	PVS1, PM2_Supporting
c.465del	p.(Phe155LeufsTer22)	3.98.E-06	0	0	0	0	0	0	3.27.E-05	0	PVS1, PM2_Supporting
c.487_488del	p.(Val163LeufsTer4)	3.98.E-06	0	0	0	0	0	8.79.E-06	0	0	PVS1, PM2_Supporting
c.509_510del	p.(Arg170IlefsTer14)	3.58.E-05	0	0	0	0	0	7.03.E-05	0	1.63.E-04	PVS1, PP1
c.599del	p.(Leu200Ter)	3.98.E-06	0	0	0	0	0	8.79.E-06	0	0	PVS1, PM2_Supporting
c.654del	p.(Asp219ThrfsTer4)	3.98.E-06	0	0	0	0	0	8.80.E-06	0	0	PVS1, PM2_Supporting
c.712A>T	p.(Arg238Ter)	3.98.E-06	0	0	0	0	0	8.80.E-06	0	0	PVS1, PM2_Supporting
c.726_727insTTAGGTCTGTAGA	p.(Thr243LeufsTer4)	3.98.E-06	0	0	0	0	0	8.80.E-06	0	0	PVS1, PM2_Supporting
c.734_735dup	p.(Thr246ArgfsTer34)	3.98.E-06	0	0	0	0	0	0	0	1.64.E-04	PVS1, PM2_Supporting
c.758dup	p.(Ser254IlefsTer3)	2.39.E-05	0	0	0	0	0	5.28.E-05	0	0	PVS1, PM2_Supporting

c.757_758del	p.(Leu253IlefsTer3)	7.96.E-06	0	0	0	0	0	1.76.E-05	0	0	PVS1, PM2_Supporting
c.886del	p.(Met296Ter)	3.98.E-06	0	0	0	0	0	8.80.E-06	0	0	PVS1, PM2_Supporting
c.979_980insATGA	p.(Cys327TyrfsTer6)	3.99.E-06	5.44.E-05	0	0	0	0	0	0	0	PVS1, PM2_Supporting
c.1010T>A	p.(Leu337Ter)	3.99.E-06	0	0	0	0	0	0	3.27.E-05	0	PVS1, PM2_Supporting
c.1050_1053del	p.(Thr351ArgfsTer4)	7.96.E-06	5.44.E-05	6.16.E-05	0	0	0	0	0	0	PVS1, PM2_Supporting
c.1056_1057del	p.(Lys353IlefsTer7)	3.98.E-06	0	0	0	0	0	0	0	1.63.E-04	PVS1, PM2_Supporting
c.1060del	p.(Ser354LeufsTer2)	3.98.E-06	0	0	0	9.92.E-05	0	0	0	0	PVS1, PM2_Supporting
c.1059_1075del	p.(Ser354Ter)	3.98.E-06	0	0	0	0	0	8.80.E-06	0	0	PVS1, PM2_Supporting
c.1120_1123del	p.(Ile374Ter)	3.98.E-06	0	0	0	0	0	8.80.E-06	0	0	PVS1, PM2_Supporting
c.1140_1143del	p.(Ser380ArgfsTer43)	7.96.E-06	0	0	0	0	0	1.76.E-05	0	0	PVS1, PM2_Supporting
c.1240C>T	p.(Arg414Ter)	7.98.E-06	0	0	2.89.E-05	0	0	8.82.E-06	0	0	PVS1, PM2_Supporting
c.1317del	p.(Phe440LeufsTer12)	4.00.E-06	0	0	0	0	0	8.85.E-06	0	0	PVS1, PM2_Supporting
c.1455_1456del	p.(Lys486SerfsTer9)	3.98.E-06	0	0	2.89.E-05	0	0	0	0	0	PVS1, PM2_Supporting
c.1479del	p.(Thr494LeufsTer67)	1.19.E-05	0	1.85.E-04	0	0	0	0	0	0	PVS1, PM2_Supporting
c.1592del	p.(Leu531CysfsTer30)	1.83.E-04	0	0	0	0	2.03.E-03	8.79.E-06	0	1.63.E-04	PVS1, PS4

c.1616_1617dup	p.(Asn540LeufsTer22)	3.98.E-06	0	0	0	0	0	8.79.E-06	0	0	PVS1, PM2_Supporting
c.1633G>T	p.(Glu545Ter)	3.98.E-06	0	0	0	0	0	0	3.27.E-05	0	PVS1, PM2_Supporting
c.1642_1643del	p.(Ser548ThrfsTer29)	3.98.E-06	0	0	0	0	0	0	3.27.E-05	0	PVS1, PM2_Supporting
c.1665dup	p.(Leu556IlefsTer22)	3.99.E-06	0	0	0	0	0	8.83.E-06	0	0	PVS1, PM2_Supporting
c.1671_1674del	p.(Ile558LysfsTer2)	4.00.E-06	0	0	0	0	0	8.87.E-06	0	0	PVS1, PM2_Supporting
c.1675C>T	p.(Gln559Ter)	8.00.E-06	0	0	5.81.E-05	0	0	0	0	0	PVS1, PM2_Supporting
c.1685-2A>G	p.(?)	4.22.E-06	0	0	0	0	0	9.52.E-06	0	0	PVS1, PM2_Supporting
c.1919C>A	p.(Ser640Ter)	3.99.E-06	0	0	0	0	0	8.81.E-06	0	0	PVS1, PM2_Supporting
c.1924del	p.(Met642CysfsTer18)	3.99.E-06	0	0	2.89.E-05	0	0	0	0	0	PVS1, PM2_Supporting
c.2006del	p.(Glu669GlyfsTer3)	3.98.E-06	0	0	0	0	0	8.79.E-06	0	0	PVS1, PM2_Supporting
c.2012T>G	p.(Leu671Ter)	7.95.E-06	0	0	0	0	0	0	6.53.E-05	0	PVS1, PM2_Supporting
c.2052del	p.(Arg686GlyfsTer23)	7.95.E-06	0	0	0	0	0	1.76.E-05	0	0	PVS1, PM2_Supporting
c.2074C>T	p.(Gln692Ter)	3.98.E-06	0	0	0	0	0	8.79.E-06	0	0	PVS1, PM2_Supporting
c.2108T>G	p.(Leu703Ter)	7.95.E-06	5.44.E-05	0	0	0	0	8.79.E-06	0	0	PVS1, PM2_Supporting
c.2120del	p.(Pro707LeufsTer2)	7.95.E-06	0	1.23.E-04	0	0	0	0	0	0	PVS1, PM2_Supporting

c.2167_2168del	p.(Met723ValfsTer21)	6.36.E-05	0	0	3.47.E-04	0	0	1.76.E-05	0	3.26.E-04	PVS1, PP1
c.2257C>T	p.(Arg753Ter)	2.39.E-05	0	0	0	0	0	4.40.E-05	0	1.63.E-04	PVS1, PM2_Supporting
c.2267_2283dup	p.(His762AlafsTer8)	3.98.E-06	0	0	0	0	0	8.79.E-06	0	0	PVS1, PM2_Supporting
c.2323C>T	p.(Gln775Ter)	3.98.E-06	0	0	0	0	0	8.79.E-06	0	0	PVS1, PM2_Supporting
c.2336C>G	p.(Ser779Ter)	1.19.E-05	0	0	8.68.E-05	0	0	0	0	0	PVS1, PM2_Supporting
c.2386G>T	p.(Gly796Ter)	3.98.E-06	0	0	0	0	0	8.79.E-06	0	0	PVS1, PM2_Supporting
c.2411_2412del	p.(Ser804CysfsTer10)	1.59.E-05	0	0	1.16.E-04	0	0	0	0	0	PVS1, PM2_Supporting
c.2488del	p.(Glu830SerfsTer21)	3.98.E-06	0	0	0	0	0	0	3.27.E-05	0	PVS1, PM2_Supporting
c.2498_2505del	p.(Lys833SerfsTer5)	3.99.E-06	0	0	0	0	0	0	3.27.E-05	0	PVS1, PM2_Supporting
c.2514+2T>A	p.(?)	4.00.E-06	0	0	0	0	0	0	3.27.E-05	0	PVS1, PM2_Supporting
c.2515-1G>C	p.(?)	3.98.E-06	0	0	2.89.E-05	0	0	0	0	0	PVS1_Moderate, PS1, PM2_Supporting, PP1
c.2559C>T	p.(Gly853=)	7.96.E-06	0	0	0	0	0	1.76.E-05	0	0	PS3, PM2_Supporting, PP3
c.2566C>T	p.(Gln856Ter)	3.98.E-06	5.44.E-05	0	0	0	0	0	0	0	PVS1, PM2_Supporting
c.2585del	p.(Lys862ArgfsTer9)	3.98.E-06	0	0	0	0	0	0	3.27.E-05	0	PVS1, PM2_Supporting
c.2586+1G>T	p.(?)	3.98.E-06	0	0	0	0	0	0	3.27.E-05	0	PVS1_Moderate, PS1, PM2_Supporting, PP1

c.2587-1G>C	p.(?)	3.98.E-06	0	0	0	0	0	0	3.27.E-05	0	PVS1_Moderate, PS3_Supporting, PM2_Supporting, PP1
c.2607del	p.(Val870Ter)	3.98.E-06	5.44.E-05	0	0	0	0	0	0	0	PVS1, PM2_Supporting
c.2727_2728del	p.(Thr911LeufsTer16)	3.98.E-06	0	0	0	0	0	8.79.E-06	0	0	PVS1, PM2_Supporting
c.2730T>A	p.(Tyr910Ter)	7.95.E-06	0	1.23.E-04	0	0	0	0	0	0	PVS1, PM2_Supporting
c.2748+1G>T	p.(?)	3.98.E-06	0	0	0	0	0	8.79.E-06	0	0	PVS1_Moderate, , PS1, PM2_Supporting, PP1
c.2748+2dup	p.(?)	3.98.E-06	0	0	0	0	0	8.79.E-06	0	0	PVS1_Moderate, PS1, PS3_Supporting, PM2_Supporting
c.2749-1G>C	p.(?)	4.01.E-06	5.45.E-05	0	0	0	0	0	0	0	PVS1, PM2_Supporting
c.2888del	p.(Ser963LeufsTer4)	3.98.E-06	0	6.15.E-05	0	0	0	0	0	0	PVS1, PM2_Supporting
c.2931dup	p.(Val978SerfsTer2)	3.98.E-06	0	0	0	0	0	8.79.E-06	0	0	PVS1, PM2_Supporting
c.2968G>T	p.(Glu990Ter)	7.95.E-06	1.09.E-04	0	0	0	0	0	0	0	PVS1, PM2_Supporting
c.3113G>A	p.(Trp1038Ter)	5.97.E-05	0	1.23.E-04	0	0	0	1.14.E-04	0	0	PVS1, PS3
c.3116del	p.(Asn1039IlefsTer2)	1.20.E-05	0	0	0	0	0	2.66.E-05	0	0	PVS1, PM2_Supporting
c.3247_3248insT	p.(Glu1083ValfsTer14)	3.98.E-06	0	0	0	0	0	8.79.E-06	0	0	PVS1, PM2_Supporting, PP1

c.3256C>T	p.(Arg1086Ter)	1.99.E-05	0	6.15.E-05	0	0	0	3.52.E-05	0	0	PVS1, PM2_Supporting, PP1
c.3298_3305dup	p.(Ser1102ArgfsTer7)	3.98.E-06	0	0	0	0	0	0	3.27.E-05	0	PVS1, PM2_Supporting, PP1
c.3324C>G	p.(Tyr1108Ter)	3.98.E-06	0	0	0	0	0	8.79.E-06	0	0	PVS1_Strong, PM2_Supporting, PP1
c.3350+4A>G	p.(?)	3.98.E-06	0	0	0	0	0	8.80.E-06	0	0	PS3, PM2_Supporting, PP1
c.3350+5G>A	p.(?)	7.96.E-06	0	0	5.78.E-05	0	0	0	0	0	PS3, PM2_Supporting, PP1
c.3362del	p.(Gly1121ValfsTer3)	3.98.E-06	0	0	0	0	0	8.80.E-06	0	0	PVS1_Strong, PM2_Supporting, PP1
c.3426_3429del	p.(Leu1142PhefsTer20)	3.98.E-06	0	0	0	0	0	8.79.E-06	0	0	PVS1_Strong, PM2_Supporting, PP1
c.3549C>A	p.(Tyr1183Ter)	3.98.E-06	0	0	0	0	0	8.79.E-06	0	0	PVS1_Strong, PM2_Supporting, PP1
c.3549C>G	p.(Tyr1183Ter)	1.59.E-05	0	0	0	0	0	3.52.E-05	0	0	PVS1_Strong, PM2_Supporting, PP1

Abbreviations: ACMG/AMP, 2015 American College of Medical Genetics and Genomics and the Association for Molecular Pathology guideline; gnomAD, Genome Aggregation Database.

Table S2. Pathogenic and likely pathogenic variants in ClinVar from gnomAD.

Nucleotide change	Amino Acid Change	gnomAD allele frequency									ACMG/AMP	ACMG/AMP evidences	HGMD
		Total	East Asain	African	Latino	Ashkenazi Jewish	European (Finnish)	European (non-Finnish)	South Asian	Other			
		(*n* = 125,748)	(*n* = 9197)	(*n* = 8128)	(*n* = 17,296)	(*n* = 5040)	(*n*=10,824)	(*n* = 56,885)	(*n* = 15,308)	(*n* = 3070)			
c.1A>G	p.(Met1?)	4.06.E-06	0	0	0	0	0	0	0	1.66.E-04	LPV	PVS1_Moderate, PS1, PM2_Supporting, PP1	
c.12dup	p.(Pro5SerfsTer8)	4.04.E-06	0	0	0	0	0	0	3.28.E-05	0	LPV	PVS1, PM2_Supporting	DM
c.62T>G	p.(Leu21Ter)	7.95.E-06	0	0	5.78.E-05	0	0	0	0	0	LPV	PVS1, PM2_Supporting	DM
c.73A>T	p.(Lys25Ter)	3.98.E-06	0	0	0	0	0	0	3.27.E-05	0	LPV	PVS1, PM2_Supporting	DM
c.79G>T	p.(Glu27Ter)	7.95.E-06	0	1.23.E-04	0	0	0	0	0	0	LPV	PVS1, PM2_Supporting	
c.172_175del	p.(Gln60ArgfsTer7)	3.98.E-05	0	0	0	9.92.E-05	0	7.91.E-05	0	0	LPV	PVS1, PP1	DM
c.178C>T	p.(Gln60Ter)	3.98.E-06	0	0	0	0	0	8.79.E-06	0	0	LPV	PVS1, PM2_Supporting	
c.196C>T	p.(Gln66Ter)	7.95.E-06	0	0	0	0	0	1.76.E-05	0	0	LPV	PVS1, PM2_Supporting	DM
c.212-2A>G	p.(?)	4.52.E-06	0	0	0	0	0	1.02.E-05	0	0	LPV	PVS1_Strong, PM2_Supporting, PP1	DM
c.226del	p.(Ile76TyrfsTer101)	4.09.E-06	0	6.31.E-05	0	0	0	0	0	0	LPV	PVS1, PM2_Supporting	DM
c.232_233del	p.(Val78LeufsTer2)	4.04.E-06	0	6.23.E-05	0	0	0	0	0	0	LPV	PVS1, PM2_Supporting	
c.395del	p.(Val132AlafsTer45)	7.98.E-06	0	0	0	0	0	1.76.E-05	0	0	LPV	PVS1, PM2_Supporting	DM
c.424A>T	p.(Lys142Ter)	7.96.E-06	0	0	0	0	0	1.76.E-05	0	0	LPV	PVS1, PM2_Supporting	DM
c.487_488del	p.(Val163LeufsTer4)	3.98.E-06	0	0	0	0	0	8.79.E-06	0	0	LPV	PVS1, PM2_Supporting	DM
c.509_510del	p.(Arg170IlefsTer14)	3.58.E-05	0	0	0	0	0	7.03.E-05	0	1.63.E-04	LPV	PVS1, PP1	DM
c.599del	p.(Leu200Ter)	3.98.E-06	0	0	0	0	0	8.79.E-06	0	0	LPV	PVS1, PM2_Supporting	DM
c.654del	p.(Asp219ThrfsTer4)	3.98.E-06	0	0	0	0	0	8.80.E-06	0	0	LPV	PVS1, PM2_Supporting	DM
c.712A>T	p.(Arg238Ter)	3.98.E-06	0	0	0	0	0	8.80.E-06	0	0	LPV	PVS1, PM2_Supporting	DM

c.726_727insTTAGGTCTGTAGA	p.(Thr243LeufsTer4)	3.98.E-06	0	0	0	0	0	8.80.E-06	0	0	LPV	PVS1, PM2_Supporting	
c.734_735dup	p.(Thr246ArgfsTer34)	3.98.E-06	0	0	0	0	0	0	0	1.64.E-04	LPV	PVS1, PM2_Supporting	
c.758dup	p.(Ser254IlefsTer3)	2.39.E-05	0	0	0	0	0	5.28.E-05	0	0	LPV	PVS1, PM2_Supporting	DM
c.757_758del	p.(Leu253IlefsTer3)	7.96.E-06	0	0	0	0	0	1.76.E-05	0	0	LPV	PVS1, PM2_Supporting	DM
c.886del	p.(Met296Ter)	3.98.E-06	0	0	0	0	0	8.80.E-06	0	0	LPV	PVS1, PM2_Supporting	DM
c.1010T>A	p.(Leu337Ter)	3.99.E-06	0	0	0	0	0	0	3.27.E-05	0	LPV	PVS1, PM2_Supporting	DM
c.1050_1053del	p.(Thr351ArgfsTer4)	7.96.E-06	5.44.E-05	6.16.E-05	0	0	0	0	0	0	LPV	PVS1, PM2_Supporting	DM
c.1056_1057del	p.(Lys353IlefsTer7)	3.98.E-06	0	0	0	0	0	0	0	1.63.E-04	LPV	PVS1, PM2_Supporting	DM
c.1140_1143del	p.(Ser380ArgfsTer43)	7.96.E-06	0	0	0	0	0	1.76.E-05	0	0	LPV	PVS1, PM2_Supporting	DM
c.1240C>T	p.(Arg414Ter)	7.98.E-06	0	0	2.89.E-05	0	0	8.82.E-06	0	0	LPV	PVS1, PM2_Supporting	DM
c.1317del	p.(Phe440LeufsTer12)	4.00.E-06	0	0	0	0	0	8.85.E-06	0	0	LPV	PVS1, PM2_Supporting	DM
c.1479del	p.(Thr494LeufsTer67)	1.19.E-05	0	1.85.E-04	0	0	0	0	0	0	LPV	PVS1, PM2_Supporting	DM
c.1592del	p.(Leu531CysfsTer30)	1.83.E-04	0	0	0	0	2.03.E-03	8.79.E-06	0	1.63.E-04	PV	PVS1, PS4	DM
c.1616_1617dup	p.(Asn540LeufsTer22)	3.98.E-06	0	0	0	0	0	8.79.E-06	0	0	LPV	PVS1, PM2_Supporting	DM
c.1633G>T	p.(Glu545Ter)	3.98.E-06	0	0	0	0	0	0	3.27.E-05	0	LPV	PVS1, PM2_Supporting	DM
c.1642_1643del	p.(Ser548ThrfsTer29)	3.98.E-06	0	0	0	0	0	0	3.27.E-05	0	LPV	PVS1, PM2_Supporting	
c.1671_1674del	p.(Ile558LysfsTer2)	4.00.E-06	0	0	0	0	0	8.87.E-06	0	0	LPV	PVS1, PM2_Supporting	DM
c.1675C>T	p.(Gln559Ter)	8.00.E-06	0	0	5.81.E-05	0	0	0	0	0	LPV	PVS1, PM2_Supporting	
c.1685-2A>G	p.(?)	4.22.E-06	0	0	0	0	0	9.52.E-06	0	0	LPV	PVS1, PM2_Supporting	DM
c.1919C>A	p.(Ser640Ter)	3.99.E-06	0	0	0	0	0	8.81.E-06	0	0	LPV	PVS1, PM2_Supporting	DM
c.1924del	p.(Met642CysfsTer18)	3.99.E-06	0	0	2.89.E-05	0	0	0	0	0	LPV	PVS1, PM2_Supporting	DM
c.2006del	p.(Glu669GlyfsTer3)	3.98.E-06	0	0	0	0	0	8.79.E-06	0	0	LPV	PVS1, PM2_Supporting	DM
c.2012T>G	p.(Leu671Ter)	7.95.E-06	0	0	0	0	0	0	6.53.E-05	0	LPV	PVS1, PM2_Supporting	DM
c.2052del	p.(Arg686GlyfsTer23)	7.95.E-06	0	0	0	0	0	1.76.E-05	0	0	LPV	PVS1, PM2_Supporting	DM
c.2074C>T	p.(Gln692Ter)	3.98.E-06	0	0	0	0	0	8.79.E-06	0	0	LPV	PVS1, PM2_Supporting	DM

c.2108T>G	p.(Leu703Ter)	7.95.E-06	5.44.E-05	0	0	0	0	8.79.E-06	0	0	LPV	PVS1, PM2_Supporting	DM
c.2120del	p.(Pro707LeufsTer2)	7.95.E-06	0	1.23.E-04	0	0	0	0	0	0	LPV	PVS1, PM2_Supporting	DM
c.2167_2168del	p.(Met723ValfsTer21)	6.36.E-05	0	0	3.47.E-04	0	0	1.76.E-05	0	3.26.E-04	LPV	PVS1, PP1	DM
c.2257C>T	p.(Arg753Ter)	2.39.E-05	0	0	0	0	0	4.40.E-05	0	1.63.E-04	LPV	PVS1, PM2_Supporting	DM
c.2267_2283dup	p.(His762AlafsTer8)	3.98.E-06	0	0	0	0	0	8.79.E-06	0	0	LPV	PVS1, PM2_Supporting	DM
c.2323C>T	p.(Gln775Ter)	3.98.E-06	0	0	0	0	0	8.79.E-06	0	0	LPV	PVS1, PM2_Supporting	DM
c.2336C>G	p.(Ser779Ter)	1.19.E-05	0	0	8.68.E-05	0	0	0	0	0	LPV	PVS1, PM2_Supporting	DM
c.2386G>T	p.(Gly796Ter)	3.98.E-06	0	0	0	0	0	8.79.E-06	0	0	LPV	PVS1, PM2_Supporting	DM
c.2411_2412del	p.(Ser804CysfsTer10)	1.59.E-05	0	0	1.16.E-04	0	0	0	0	0	LPV	PVS1, PM2_Supporting	DM
c.2488del	p.(Glu830SerfsTer21)	3.98.E-06	0	0	0	0	0	0	3.27.E-05	0	LPV	PVS1, PM2_Supporting	DM
c.2498_2505del	p.(Lys833SerfsTer5)	3.99.E-06	0	0	0	0	0	0	3.27.E-05	0	LPV	PVS1, PM2_Supporting	
c.2515-1G>C	p.(?)	3.98.E-06	0	0	2.89.E-05	0	0	0	0	0	LPV	PVS1_Moderate, PS1, PM2_Supporting, PP1	
c.2559C>T	p.(Gly853=)	7.96.E-06	0	0	0	0	0	1.76.E-05	0	0	LPV	PS3, PM2_Supporting, PP3	DM
c.2566C>T	p.(Gln856Ter)	3.98.E-06	5.44.E-05	0	0	0	0	0	0	0	LPV	PVS1, PM2_Supporting	
c.2585del	p.(Lys862ArgfsTer9)	3.98.E-06	0	0	0	0	0	0	3.27.E-05	0	LPV	PVS1, PM2_Supporting	
c.2586+1G>T	p.(?)	3.98.E-06	0	0	0	0	0	0	3.27.E-05	0	LPV	PVS1_Moderate, PS1, PM2_Supporting, PP1	
c.2607del	p.(Val870Ter)	3.98.E-06	5.44.E-05	0	0	0	0	0	0	0	LPV	PVS1, PM2_Supporting	DM
c.2727_2728del	p.(Thr911LeufsTer16)	3.98.E-06	0	0	0	0	0	8.79.E-06	0	0	LPV	PVS1, PM2_Supporting	DM
c.2730T>A	p.(Tyr910Ter)	7.95.E-06	0	1.23.E-04	0	0	0	0	0	0	LPV	PVS1, PM2_Supporting	DM
c.2748+1G>T	p.(?)	3.98.E-06	0	0	0	0	0	8.79.E-06	0	0	LPV	PVS1_Moderate, , PS1, PM2_Supporting, PP1	DM
c.2749-1G>C	p.(?)	4.01.E-06	5.45.E-05	0	0	0	0	0	0	0	LPV	PVS1, PM2_Supporting	DM
c.2888del	p.(Ser963LeufsTer4)	3.98.E-06	0	6.15.E-05	0	0	0	0	0	0	LPV	PVS1, PM2_Supporting	DM

c.2931dup	p.(Val978SerfsTer2)	3.98.E-06	0	0	0	0	0	8.79.E-06	0	0	LPV	PVS1, PM2_Supporting	DM
c.2968G>T	p.(Glu990Ter)	7.95.E-06	1.09.E-04	0	0	0	0	0	0	0	LPV	PVS1, PM2_Supporting	DM
c.3113G>A	p.(Trp1038Ter)	5.97.E-05	0	1.23.E-04	0	0	0	1.14.E-04	0	0	PV	PVS1, PS3	DM
c.3116del	p.(Asn1039IlefsTer2)	1.20.E-05	0	0	0	0	0	2.66.E-05	0	0	LPV	PVS1, PM2_Supporting	DM
c.3247_3248insT	p.(Glu1083ValfsTer14)	3.98.E-06	0	0	0	0	0	8.79.E-06	0	0	PV	PVS1, PM2_Supporting, PP1	
c.3256C>T	p.(Arg1086Ter)	1.99.E-05	0	6.15.E-05	0	0	0	3.52.E-05	0	0	PV	PVS1, PM2_Supporting, PP1	DM
c.3298_3305dup	p.(Ser1102ArgfsTer7)	3.98.E-06	0	0	0	0	0	0	3.27.E-05	0	PV	PVS1, PM2_Supporting, PP1	
c.3324C>G	p.(Tyr1108Ter)	3.98.E-06	0	0	0	0	0	8.79.E-06	0	0	LPV	PVS1_Strong, PM2_Supporting, PP1	DM
c.3350+4A>G	p.(?)	3.98.E-06	0	0	0	0	0	8.80.E-06	0	0	LPV	PS3, PM2_Supporting, PP1	DM
c.3350+5G>A	p.(?)	7.96.E-06	0	0	5.78.E-05	0	0	0	0	0	LPV	PS3, PM2_Supporting, PP1	DM
c.3362del	p.(Gly1121ValfsTer3)	3.98.E-06	0	0	0	0	0	8.80.E-06	0	0	LPV	PVS1_Strong, PM2_Supporting, PP1	DM
c.3426_3429del	p.(Leu1142PhefsTer20)	3.98.E-06	0	0	0	0	0	8.79.E-06	0	0	LPV	PVS1_Strong, PM2_Supporting, PP1	DM
c.3549C>A	p.(Tyr1183Ter)	3.98.E-06	0	0	0	0	0	8.79.E-06	0	0	LPV	PVS1_Strong, PM2_Supporting, PP1	DM
c.3549C>G	p.(Tyr1183Ter)	1.59.E-05	0	0	0	0	0	3.52.E-05	0	0	LPV	PVS1_Strong, PM2_Supporting, PP1	DM

ACMG/AMP, 2015 American College of Medical Genetics and Genomics and the Association for Molecular Pathology guideline; *DM*, disease-causing variant; *gnomAD*, Genome Aggregation Database; HGMD, Human Gene Mutation Database; *LPV*, likely pathogenic variant; *PV*, pathogenic variant.

Table S3. Disease-causing mutations in the Human Gene Mutation Database (HGMD) from gnomAD.

Nucleotide change	Amino Acid Change	gnomAD Allele Frequency									ACMG/AMP	ACMG/AMP evidences	ClinVar
		Total (*n* = 125,748)	East Asain (*n* = 9197)	African (*n* = 8128)	Latino (*n* = 17,296)	Ashkenazi Jewish (*n* = 5040)	European (Finnish) (*n* = 10,824)	European (non-Finnish) (*n* = 56,885)	South Asian (*n* = 15,308)	Other (*n*=3070)			
c.2T>C	p.(Met1?)	4.06E-06	0	0	0	0	0	9.04E-06	0	0	LPV	PVS1_Moderate, PS1, PM2_Supporting	Conflict
c.12dup	p.(Pro5SerfsTer8)	4.04E-06	0	0	0	0	0	0	3.28E-05	0	LPV	PVS1, PM2_Supporting	PV
c.62T>G	p.(Leu21Ter)	7.95E-06	0	0	5.78E-05	0	0	0	0	0	LPV	PVS1, PM2_Supporting	PV
c.73A>T	p.(Lys25Ter)	3.98E-06	0	0	0	0	0	0	3.27E-05	0	LPV	PVS1, PM2_Supporting	PV
c.109-12T>A	p.(?)	3.98E-06	0	0	0	0	0	8.79E-06	0	0	LPV	PS1_Supporting, PS3_Moderate, PM2_Supporting, PP1, PP3	Conflict
c.109C>A	p.(Arg37Ser)	1.59E-05	0	1.23E-04	2.89E-05	0	0	8.79E-06	0	0	VUS	PS1_Moderate, PM2_Supporting, BP4_Supporting	VUS
c.113C>G	p.(Ala38Gly)	2.78E-05	3.81E-04	0	0	0	0	0	0	0	VUS	PM2_Supporting	VUS
c.172_175del	p.(Gln60ArgfsTer7)	3.98E-05	0	0	0	9.92E-05	0	7.91E-05	0	0	LPV	PVS1, PP1	PV/LPV
c.196C>T	p.(Gln66Ter)	7.95E-06	0	0	0	0	0	1.76E-05	0	0	LPV	PVS1, PM2_Supporting	PV/LPV
c.212-2A>G	p.(?)	4.52E-06	0	0	0	0	0	1.02E-05	0	0	LPV	PVS1_Strong, PM2_Supporting, PP1	PV/LPV

c.226del	p.(Ile76TyrfsTer101)	4.09E-06	0	6.31E-05	0	0	0	0	0	0	LPV	PVS1, PM2_Supporting	PV/LPV
c.395del	p.(Val132AlafsTer45)	7.98E-06	0	0	0	0	0	1.76E-05	0	0	LPV	PVS1, PM2_Supporting	PV
c.424A>T	p.(Lys142Ter)	7.96E-06	0	0	0	0	0	1.76E-05	0	0	LPV	PVS1, PM2_Supporting	PV
c.487_488del	p.(Val163LeufsTer4)	3.98E-06	0	0	0	0	0	8.79E-06	0	0	LPV	PVS1, PM2_Supporting	PV
c.509_510del	p.(Arg170IlefsTer14)	3.58E-05	0	0	0	0	0	7.03E-05	0	1.63E-04	LPV	PVS1, PP1	PV
c.599del	p.(Leu200Ter)	3.98E-06	0	0	0	0	0	8.79E-06	0	0	LPV	PVS1, PM2_Supporting	PV
c.654del	p.(Asp219ThrfsTer4)	3.98E-06	0	0	0	0	0	8.80E-06	0	0	LPV	PVS1, PM2_Supporting	PV
c.712A>T	p.(Arg238Ter)	3.98E-06	0	0	0	0	0	8.80E-06	0	0	LPV	PVS1, PM2_Supporting	PV
c.758dup	p.(Ser254IlefsTer3)	2.39E-05	0	0	0	0	0	5.28E-05	0	0	LPV	PVS1, PM2_Supporting	PV/LPV
c.757_758del	p.(Leu253IlefsTer3)	7.96E-06	0	0	0	0	0	1.76E-05	0	0	LPV	PVS1, PM2_Supporting	PV
c.886del	p.(Met296Ter)	3.98E-06	0	0	0	0	0	8.80E-06	0	0	LPV	PVS1, PM2_Supporting	PV
c.1010T>A	p.(Leu337Ter)	3.99E-06	0	0	0	0	0	0	3.27E-05	0	LPV	PVS1, PM2_Supporting	PV
c.1050_1053del	p.(Thr351ArgfsTer4)	7.96E-06	5.44E-05	6.16E-05	0	0	0	0	0	0	LPV	PVS1, PM2_Supporting	PV
c.1056_1057del	p.(Lys353IlefsTer7)	3.98E-06	0	0	0	0	0	0	0	1.63E-04	LPV	PVS1, PM2_Supporting	PV
c.1140_1143del	p.(Ser380ArgfsTer43)	7.96E-06	0	0	0	0	0	1.76E-05	0	0	LPV	PVS1, PM2_Supporting	PV
c.1240C>T	p.(Arg414Ter)	7.98E-06	0	0	2.89E-05	0	0	8.82E-06	0	0	LPV	PVS1, PM2_Supporting	PV

c.1317del	p.(Phe440LeufsTer12)	4.00E-06	0	0	0	0	0	8.85E-06	0	0	LPV	PVS1, PM2_Supporting	PV
c.1479del	p.(Thr494LeufsTer67)	1.19E-05	0	1.85E-04	0	0	0	0	0	0	LPV	PVS1, PM2_Supporting	PV
c.1592del	p.(Leu531CysfsTer30)	1.83E-04	0	0	0	0	2.03E-03	8.79E-06	0	1.63E-04	PV	PVS1, PS4	PV
c.1616_1617dup	p.(Asn540LeufsTer22)	3.98E-06	0	0	0	0	0	8.79E-06	0	0	LPV	PVS1, PM2_Supporting	PV
c.1633G>T	p.(Glu545Ter)	3.98E-06	0	0	0	0	0	0	3.27E-05	0	LPV	PVS1, PM2_Supporting	PV
c.1671_1674del	p.(Ile558LysfsTer2)	4.00E-06	0	0	0	0	0	8.87E-06	0	0	LPV	PVS1, PM2_Supporting	PV
c.1685-2A>G	p.(?)	4.22E-06	0	0	0	0	0	9.52E-06	0	0	LPV	PVS1, PM2_Supporting	LPV
c.1882_1890del	p.(Lys628_Cys630del)	3.19E-05	0	0	2.89E-05	0	0	5.28E-05	0	1.64E-04	VUS	PM2_Supporting, PM4	VUS
c.1919C>A	p.(Ser640Ter)	3.99E-06	0	0	0	0	0	8.81E-06	0	0	LPV	PVS1, PM2_Supporting	PV
c.1924del	p.(Met642CysfsTer18)	3.99E-06	0	0	2.89E-05	0	0	0	0	0	LPV	PVS1, PM2_Supporting	PV/LPV
c.2006del	p.(Glu669GlyfsTer3)	3.98E-06	0	0	0	0	0	8.79E-06	0	0	LPV	PVS1, PM2_Supporting	PV
c.2012T>G	p.(Leu671Ter)	7.95E-06	0	0	0	0	0	0	6.53E-05	0	LPV	PVS1, PM2_Supporting	PV/LPV
c.2052del	p.(Arg686GlyfsTer23)	7.95E-06	0	0	0	0	0	1.76E-05	0	0	LPV	PVS1, PM2_Supporting	PV
c.2074C>T	p.(Gln692Ter)	3.98E-06	0	0	0	0	0	8.79E-06	0	0	LPV	PVS1, PM2_Supporting	LPV
c.2108T>G	p.(Leu703Ter)	7.95E-06	5.44E-05	0	0	0	0	8.79E-06	0	0	LPV	PVS1, PM2_Supporting	PV
c.2120del	p.(Pro707LeufsTer2)	7.95E-06	0	1.23E-04	0	0	0	0	0	0	LPV	PVS1, PM2_Supporting	PV

c.2167_2168del	p.(Met723ValfsTer21)	6.36E-05	0	0	3.47E-04	0	0	1.76E-05	0	3.26E-04	LPV	PVS1, PP1	PV
c.2257C>T	p.(Arg753Ter)	2.39E-05	0	0	0	0	0	4.40E-05	0	1.63E-04	LPV	PVS1, PM2_Supporting	PV
c.2267_2283dup	p.(His762AlafsTer8)	3.98E-06	0	0	0	0	0	8.79E-06	0	0	LPV	PVS1, PM2_Supporting	PV
c.2323C>T	p.(Gln775Ter)	3.98E-06	0	0	0	0	0	8.79E-06	0	0	LPV	PVS1, PM2_Supporting	PV
c.2336C>G	p.(Ser779Ter)	1.19E-05	0	0	8.68E-05	0	0	0	0	0	LPV	PVS1, PM2_Supporting	PV/LPV
c.2386G>T	p.(Gly796Ter)	3.98E-06	0	0	0	0	0	8.79E-06	0	0	LPV	PVS1, PM2_Supporting	PV/LPV
c.2411_2412del	p.(Ser804CysfsTer10)	1.59E-05	0	0	1.16E-04	0	0	0	0	0	LPV	PVS1, PM2_Supporting	PV/LPV
c.2488del	p.(Glu830SerfsTer21)	3.98E-06	0	0	0	0	0	0	3.27E-05	0	LPV	PVS1, PM2_Supporting	PV
c.2559C>T	p.(Gly853=)	7.96E-06	0	0	0	0	0	1.76E-05	0	0	LPV	PS3, PM2_Supporting, PP3	LPV
c.2587-1G>C	p.(?)	3.98E-06	0	0	0	0	0	0	3.27E-05	0	LPV	PVS1_Moderate, PS3_Supporting, PM2_Supporting, PP1	Conflict
c.2607del	p.(Val870Ter)	3.98E-06	5.44E-05	0	0	0	0	0	0	0	LPV	PVS1, PM2_Supporting	PV/LPV
c.2727_2728del	p.(Thr911LeufsTer16)	3.98E-06	0	0	0	0	0	8.79E-06	0	0	LPV	PVS1, PM2_Supporting	PV/LPV
c.2730T>A	p.(Tyr910Ter)	7.95E-06	0	1.23E-04	0	0	0	0	0	0	LPV	PVS1, PM2_Supporting	PV/LPV
c.2748+1G>T	p.(?)	3.98E-06	0	0	0	0	0	8.79E-06	0	0	LPV	PVS1_Moderate, , PS1, PM2_Supporting, PP1	PV/LPV
c.2749-1G>C	p.(?)	4.01E-06	5.45E-05	0	0	0	0	0	0	0	LPV	PVS1, PM2_Supporting	LPV

c.2753C>A	p.(Pro918Gln)	3.20E-05	0	0	2.32E-04	0	0	0	0	0	VUS	PM1, PM2_Supporting	Conflict
c.2888del	p.(Ser963LeufsTer4)	3.98E-06	0	6.15E-05	0	0	0	0	0	0	LPV	PVS1, PM2_Supporting	PV
c.2931dup	p.(Val978SerfsTer2)	3.98E-06	0	0	0	0	0	8.79E-06	0	0	LPV	PVS1, PM2_Supporting	PV
c.2968G>T	p.(Glu990Ter)	7.95E-06	1.09E-04	0	0	0	0	0	0	0	LPV	PVS1, PM2_Supporting	PV
c.2978C>T	p.(Thr993Met)	1.99E-05	5.44E-05	6.15E-05	5.78E-05	0	0	8.79E-06	0	0	VUS	PM1, PM2_Supporting, BP4_Moderate	Conflict
c.2997-1G>A	p.(?)	3.98E-06	0	0	0	0	0	0	3.27E-05	0	VUS	PVS1_Moderate, PM2_Supporting	Conflict
c.3113G>A	p.(Trp1038Ter)	5.97E-05	0	1.23E-04	0	0	0	1.14E-04	0	0	PV	PVS1, PS3	PV
c.3116del	p.(Asn1039IlefsTer2)	1.20E-05	0	0	0	0	0	2.66E-05	0	0	LPV	PVS1, PM2_Supporting	PV/LPV
c.3256C>T	p.(Arg1086Ter)	1.99E-05	0	6.15E-05	0	0	0	3.52E-05	0	0	PV	PVS1, PM2_Supporting, PP1	PV/LPV
c.3324C>G	p.(Tyr1108Ter)	3.98E-06	0	0	0	0	0	8.79E-06	0	0	LPV	PVS1_Strong, PM2_Supporting, PP1	PV/LPV
c.3350+4A>G	p.(?)	3.98E-06	0	0	0	0	0	8.80E-06	0	0	LPV	PS3, PM2_Supporting, PP1	LPV
c.3350+5G>A	p.(?)	7.96E-06	0	0	5.78E-05	0	0	0	0	0	LPV	PS3, PM2_Supporting, PP1	PV/LPV
c.3362del	p.(Gly1121ValfsTer3)	3.98E-06	0	0	0	0	0	8.80E-06	0	0	LPV	PVS1_Strong, PM2_Supporting, PP1	LPV
c.3428T>A	p.(Leu1143His)	1.71E-04	0	6.15E-05	5.78E-05	9.92E-05	0	2.73E-04	1.63E-04	4.89E-04	VUS	PM1, PM5, BP4_Supporting	Conflict
c.3426_3429del	p.(Leu1142PhefsTer20)	3.98E-06	0	0	0	0	0	8.79E-06	0	0	LPV	PVS1_Strong, PM2_Supporting, PP1	PV
c.3539T>C	p.(Ile1180Thr)	3.98E-06	0	0	0	0	0	8.79E-06	0	0	VUS	PM1, PM2_Supporting	VUS

c.3549C>A	p.(Tyr1183Ter)	3.98E-06	0	0	0	0	0	8.79E-06	0	0	LPV	PVS1_Strong, PM2_Supporting, PP1	PV
c.3549C>G	p.(Tyr1183Ter)	1.59E-05	0	0	0	0	0	3.52E-05	0	0	LPV	PVS1_Strong, PM2_Supporting, PP1	PV/LPV

ACMG/AMP, 2015 American College of Medical Genetics and Genomics and the Association for Molecular Pathology guideline; Conflict, Conflicting classifications of pathogenicity; *DM*, disease-causing variant; *gnomAD*, Genome Aggregation Database; HGMD, Human Gene Mutation Database; *LPV*, likely pathogenic variant; *PV*, pathogenic variant; VUS, variants of uncertain significance.

Table S4. Pathogenic and likely pathogenic variants in Korean databases classified according to the ACMG/AMP guidelines.

Nucleotide change	Amino Acid Change	Korean Database Allele Frequency			gnomAD Allele Frequency									ACMG/AMP evidences
		gnomAD Korean (*n* = 1909)	KOVA (*n* = 5305)	KRGDB (*n* = 1722)	Total (*n* = 125,748)	East Asian (*n* = 9197)	African (*n* = 8128)	Latino (*n* = 17,296)	Ashkenazi Jewish (*n* = 5040)	European (Finnish) (*n* = 10,824)	European (non-Finnish) (*n* = 56,885)	South Asian (*n* = 15,308)	Other (*n* = 3,070)	
c.228_229del	p.(Ile76MetfsTer4)	0	1.50.E-04	0	0	0	0	0	0	0	0	0	0	PVS1, PM2_Supporting
c.695del	p.(Gly232ValfsTer6)	0	1.47.E-04	0	0	0	0	0	0	0	0	0	0	PVS1, PM2_Supporting, PP1
c.964G>T	p.(Glu322Ter)	0	1.47.E-04	0	0	0	0	0	0	0	0	0	0	PVS1, PM2_Supporting
c.979_980insATGA	p.(Cys327TyrfsTer6)	2.62.E-04	0	0	3.99.E-06	5.44.E-05	0	0	0	0	0	0	0	PVS1, PM2_Supporting
c.1048C>T	p.(Gln350Ter)	0	2.83.E-04	0	0	0	0	0	0	0	0	0	0	PVS1, PM2_Supporting, PP1
c.1804C>T	p.(Gln602Ter)	0	1.47.E-04	0	0	0	0	0	0	0	0	0	0	PVS1, PM2_Supporting
c.2167_2168del	p.(Met723ValfsTer21)	0	1.47.E-04	0	6.36.E-05	0	0	3.47.E-04	0	0	1.76.E-05	0	3.26.E-04	PVS1, PP1
c.2566C>T	p.(Gln856Ter)	2.62.E-04	0	0	3.98.E-06	5.44.E-05	0	0	0	0	0	0	0	PVS1, PM2_Supporting
c.2834+2T>C	p.(?)	0	1.48.E-04	0	0	0	0	0	0	0	0	0	0	PVS1, PM2_Supporting
c.3267_3268del	p.(Phe1090SerfsTer6)	0	2.68.E-04	0	0	0	0	0	0	0	0	0	0	PVS1, PM2_Supporting

ACMG/AMP, 2015 American College of Medical Genetics and Genomics and the Association for Molecular Pathology guideline; *gnomAD*, Genome Aggregation Database; KOVA, Korean Variant Archive; KRGDB, Korean Reference Genome Database.
